# Designing Urban Green Spaces for Older Adults in Asian Cities

**DOI:** 10.3390/ijerph16224423

**Published:** 2019-11-12

**Authors:** Zheng Tan, Kevin Ka-Lun Lau, Adam Charles Roberts, Stessa Tzu-Yuan Chao, Edward Ng

**Affiliations:** 1 Institute of Future Cities, The Chinese University of Hong Kong, Hong Kong 999077, China; zheng.tan@yncrea.fr (Z.T.); edwardng@cuhk.edu.hk (E.N.); 2 Hautes Etudes D’ingénieur, Yncréa, Université Catholique de Lille, 59000 Lille, France; 3 Laboratoire de Génie Civil et géo-Environnement, 59000 Lille, France; 4 CUHK Jockey Club Institute of Ageing, The Chinese University of Hong Kong, Hong Kong 999077, China; 5 Institute of Environment, Energy and Sustainability, The Chinese University of Hong Kong, Hong Kong 999077, China; 6 School of Mechanical and Aerospace Engineering, Nanyang Technological University, Singapore 639798, Singapore; aroberts@ntu.edu.sg; 7 Department of Urban Planning, National Cheng Kung University, Tainan 70101, Taiwan; tychao@ncku.edu.tw; 8 School of Architecture, The Chinese University of Hong Kong, Hong Kong 999077, China

**Keywords:** age-friendly cities, urban design, self-reported health, environmental perception

## Abstract

Elderly populations in Asian countries are expected to increase rapidly in the next few decades. Older adults, particularly in high-density cities, spend a considerable amount of time in urban green spaces (UGSs). The World Health Organization noted that UGSs are key to improving the age-friendliness of neighborhoods. Thus, it is necessary to design UGSs for the promotion of healthy ageing to enhance preventive healthcare and relieve medical burdens. This study conducted interviews using a questionnaire with a sample size of 326 participants in the cities of Hong Kong (China) and Tainan (Taiwan region). The inter-relationships among the design of UGSs (e.g., spatial distribution and accessibility, characteristics of plants and UGSs), older adults’ perceptions on safety and aesthetics quality of UGSs, and their self-reported health conditions (assessed by the self-reported SF-12v2 Health Survey) were investigated with bivariate Spearman rank correlation tests. The results indicate that the duration of visits to UGSs was positively associated with mental health and social functioning, two subscales evaluating health-related quality of life in SF 12v2. The statistical model (moderation analysis) showed that such a correlation was especially significant in women and those with low social support and social capital. A positive relationship was found between the physical health subscale and perceived safety in UGSs. This relationship was stronger among older adults living alone (moderation analysis). Furthermore, the color of plants and maintenance condition of UGSs were significant aspects affecting the subjective assessment of aesthetic quality. This study provides useful information regarding how to plan and design urban green spaces with certain characteristics that could improve the accessibility and aesthetic quality, which are preferred by older adults.

## 1. Introduction

Asian countries, particularly East Asian countries, are currently experiencing an ageing trend that is unprecedented in history. According to the World Bank report, *Live Long and Prosper: Aging in East Asia and Pacific*, there were more than 211 million elderly people (people aged 65 or above) living in East Asia and the Pacific in 2010, which accounts for approximately 36% of the global population of the same age group. This group was projected to increase by approximately 22% every five years in East Asian countries [1]. Both China and Korea have met the international standard for an ageing society (i.e., people aged 65 or older comprise over 7% of the total population) since 2000 [2]. In 2016, 10.5% of the total population in China was aged 65 and above [3]. In Hong Kong, the proportion of the elderly population is expected to exceed 30% (approximately 2.6 million) by 2041 [4]. In Taiwan, senior citizens above age 65 comprised 12% of the entire population at the end of 2014. According to the census data on ageing populations by cities, Tainan and Gaoxiong had more than 20% of the population above the age of 65, and Taibei had over 25% [5]. Taiwan will cross the threshold into a super-aged society, with one in every five people being an older adult [6].

Urban environments are becoming particularly important in promoting healthy ageing [7]. Approximately 80% of the elderly population in developed countries now live in cities, and so will 25% of the elderly population in developing countries by 2050 [8]. Urban green spaces (UGSs) are regarded as one of the essential features in creating age-friendly communities [9]. A previous study showed that elderly residents in Hong Kong spend a large amount of time in UGS [10]. The benefits of UGSs include enhancing physical and mental health, maintaining a social network, and forming a sense of belonging among older adults [11,12,13].

While studies abound on the benefits and public appreciation of UGSs, the influence of environmental characteristics on the perceived quality of UGSs among older adults has seldom been discussed [14]. Van Herzele and Wiedemann [15] concluded that duration of visits to UGSs is largely affected by perceived safety, aesthetic quality (e.g., unity, naturalness, and historic character), and accessibility of the UGS. On the other hand, studies have identified different factors associated with older adults’ perceived safety in UGSs. Some studies focused on the physical setting of the UGS, e.g., the arrangement of greenery, edge treatments, visual access, and number of visitors on the site [16,17,18]. Other studies noted that concern over security is associated with reduced mobility and a sense of frailty and vulnerability [19,20]. However, findings from these studies are based on Western countries and cross-cultural validation has not yet been done [21]. A previous study also pointed to the differences in need among older adults with varying health and socio-economic statuses [22]. Further research is therefore required for a better understanding of perceptions of different elderly groups towards UGSs.

Previous research in UGS sin Asian cities was focused on urban parks; for example, Wu and Song [23] studied the inclusive design of urban parks in Taiwan and noted that safety, accessibility, and maintenance are the key aspects for elderly visitors. However, urbanization generates significant tension in terms of vegetation cover and green spaces in Asian high-density cities [24,25]. With decreasing large urban parks in gardens in high-density cities, more research effort should be placed on the proper planning and design of small-scale green spaces in highly developed urban settings [26]. Such small-scale UGSs, according to *Hong Kong Planning Standards and Guidelines* [27], are landscaped recreational open spaces where people can enjoy natural elements in a leisurely manner. This would include street gardens, vegetated sitting-out areas, and children’s playgrounds with greenery [28]. Compared to city parks, such small-scale UGSs within easy walking distance are often more highly valued by elderly citizens [15,29]. Furthermore, D’Acci [30] pointed to the link between environment psychology, user preference, and urban design. James et al. [31] stated that the scientific understanding on urban greenery should be integrated into the planning and design processes of UGSs. For example, Barbosa et al. [11] investigated how access to UGSs varied across different age groups and highlighted the need for additional green space in neighborhood planning.

The primary objective of the present study was to address older adults’ subjective assessment of UGSs and provide useful information for UGS design for older adults, with a special focus on the following research questions:

1. What are the potential impacts of UGSs on self-reported health and well-being for older adults in Asia? Which age group benefits most from visiting UGSs?
2. Are there any differences in perceptions and preferences of UGSs among older adults with various self-reported health conditions?
3. What are the key design aspects of UGSs that affect the perceptions of older adults? How do we properly plan UGSs to promote active ageing in high-density Asian cities?

## 2. Methodology and Study Area

### 2.1. Study Area and Site Audit

**Figure 1:**
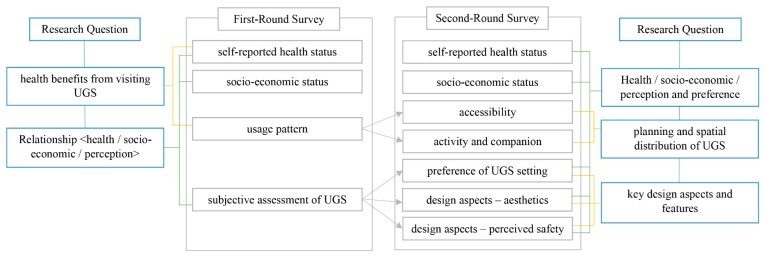
Research diagram of the study.

This study aimed to examine older adults’ perception of UGS and discuss how to plan and design UGS to facilitate their needs. Two high-density cities with rapidly ageing populations, namely Hong Kong and Tainan, were selected for the study. On-site face-to-face interviews using a traditional paper-and-pencil questionnaire were conducted, a mode of questionnaire administration that offers high accuracy and quality of the data in completion of the questionnaire, recall bias, amount of information, etc. [32]. Audits in UGSs were carried out by designers and experts for site selection [33]. Two rounds of questionnaires were conducted, with the first round as a pilot study and the second round as in-depth interviewing involving planning and design aspects. Between December 2016 and March 2018, 326 older adults were interviewed at 31 small-scale UGS sites in the two cities (see Figure 1 for the research diagram).

**Figure 2:**
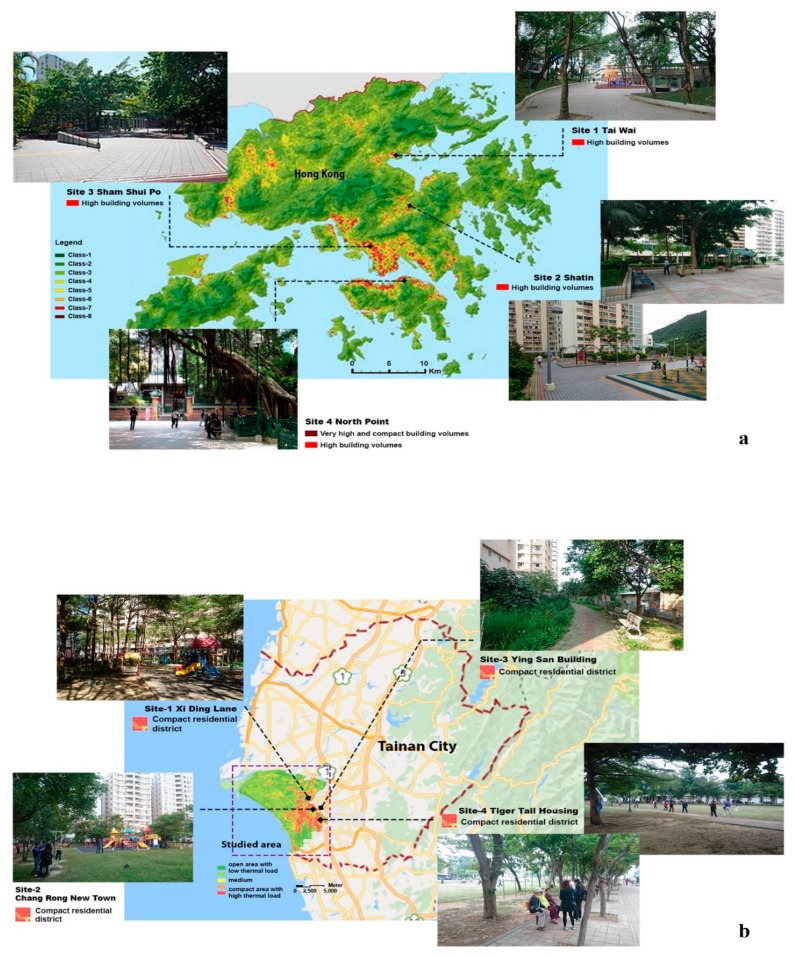
Study sites of Hong Kong (**a**) and Tainan (**b**) in the first survey (for both maps, darker colors in the legend indicate urban areas with higher density. Please refer to [39,40] for further information).

**Figure 3:**
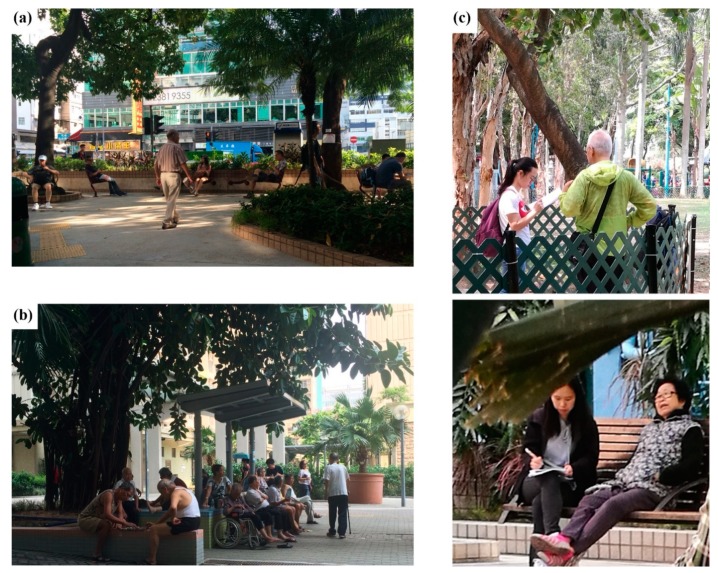
Two types of urban green spaces included in the second survey, (**a**) street gardens and (**b**) green areas in public housing estates, and (**c**) respondents taking the survey. (Photos taken with the knowledge and consent of participants.)

Hong Kong is located on the southern coast of China and has a population density of 6690 people per km^2^ [4]. The proportion of residents aged 65 years and older reached a new high of 16% in 2016 and is projected to reach 30% in 2041. Tainan is located in the southwestern region of Taiwan. Based on 2016 statistics (National Statistics, Taiwan), Tainan has a land area of 2192 km^2^, and its population density in urban areas is 4500 people per km^2^. Tainan has the second largest ageing population in Taiwan, with residents aged 65 years or older accounting for nearly 14% of Tainan’s population. Hong Kong [34] and Tainan [35,36] are good representatives of high-density cities in Asia. Small-scale UGSs (Figure 2 and Figure 3) are commonly found in the compact urban centers of the two cities [6,37,38].

Previously, questionnaire studies have been used to collect subjective data of UGSs with environmental satisfaction in urban areas [41,42] as well as the impact of UGS on health and wellbeing [43]. These subjective measures have been shown to achieve high levels of internal reliability and accuracy [44]. In addition, subjective measures are, in some ways, more relevant in investigating older adults’ perceptions and preferences towards UGSs, as objective measures may overlook or mask implicit variables of interest [45,46].

The first round of the questionnaire survey was designed as a pilot study. It was carried out in nine selected small-scale UGSs in Hong Kong and Tainan to investigate the inter-relationships among the characteristics of the UGS, older adults’ perceptions, and their self-reported health conditions. Based on the pilot, the interview instrument was refined for the second round of the survey [47]. The second round of questionnaires was designed to provide further information on possible relationships between health and socioeconomic status and older adults’ perceptions and preferences for the UGS. Before the interview took place, audits in UGSs were conducted in Hong Kong by a team formed by architects, landscape architects, and social scientists. The audits offered first-hand information on the existing design of UGSs and use patterns of older visitors. Then, based on the observed popularity among older adults, 22 small-scale UGSs in Hong Kong were selected for the second round of the survey. The interviews were carried out during the hours of 800 and 1100 and 1400 and 1600 on weekdays. From observations, older adults are much more likely to be present in UGSs during these periods of day, and there is a greater percentage of older adult visitors to UGSs on weekdays than in weekends.

### 2.2. Questionnaire Design

The questionnaire used in the study was developed from validated questionnaires from previous studies and self-designed questions (e.g., preferences for UGSs, design aspects related to aesthetic quality) [48]. Questions in the first-round survey focused on the following aspects: (1) Subjective assessment of the quality and characteristics of UGSs, (2) usage patterns, (3) self-reported health status, and (4) socio-demographic information. For the second-round survey, the questionnaire used was modified and extended to obtain specific information about the design features of UGSs. Based on the first-round survey and site audits, sub-scales were also added for the following topics: (1) Accessibility of the UGS, (2) activity in different types of UGSs, (3) preferences for UGSs (settings of greenery, view, ambience), (4) design aspects related to aesthetic quality (color, shape, and variation of plants; maintenance; proportions of hard and soft surfaces), and (5) aspects associated with perceived safety (see Table 1).

The SF12v2 has been proven to be a reliable tool for health evaluation and can properly distinguish a variety of health statuses for older adults [49,50]. In the SF12v2, respondents’ health status is measured in eight domains, including self-rated general health status, physical functioning, physical role, bodily pain, vitality, social functioning, emotional role, and mental health. Data collected from the survey were then entered into QualityMetric Health OutcomesTM Scoring Software 5.1 (QualityMetric Incorporated, Lincoln, U.S.A.)and calculated, and the scores for each respondent on the eight domains and the physical component summary (PCS) score and mental component summary (MCS) score were obtained [51].

The participants were also asked to respond to three open-ended questions on the potential negative effects of plants, and the most satisfying and unsatisfying aspects of the UGS.

Cluster sampling, as an effective and efficient means to evaluate a large population [53], was applied in this study. Older adults aged 55 years or above were chosen to include both the younger elderly population (age between 55 and 70 years) and the older elderly population (above 70 years) in the study (Figure 3c) in accordance with previous research [62,63,64,65]. Oral administration of questions was used in the study. Compared to a self-administered questionnaire, an oral interview allows better interpretation of the question and response choice for older adults with limited reading skills [66]. Samples were randomly selected on site. At the beginning of the interview, the interviewees were first asked to provide information on their age. The interview would not proceed further if the age of the interviewee was below 55 years. During the first-round survey, a total of 442 persons were invited on site to participate in the survey, and 118 in Hong Kong (39% response rate) and 99 in Tainan (71% response rate) completed the questionnaire. For the second-round survey in Hong Kong, 180 persons were invited, with 109 successful responses obtained. All participants participated in this study voluntarily after giving informed consent under the protocols approved by the Survey Ethics Committee of the Chinese University of Hong Kong. The most commonly reported reasons for not participating in the interview included that it was time-consuming and concerns over a lack of relevant knowledge.

### 2.3. Statistical Analysis

The acquired data were then included in subsequent statistical analyses, with bivariate Spearman rank correlation tests (a method to measure the strength and direction of the association between two continuous or ordinal variables [67,68] used to investigate the correlations among characteristics of the UGS, self-reported health status, and older adults’ perceptions of and preferences for the UGS [69]. A linear model was used to identify key design aspects related to subjective assessment of the aesthetic quality of the UGS. This model was also applied to investigate the key factors contributing to perceptions of safety. Moderation analysis, a model assessing how much the strength (and direction) of the relationship between dependent and independent variables is affected by a third variable [70], has been widely employed in behavioral studies to investigate the influence of personal factors [71,72,73]. Following the same method, this study performed moderated regression analyses and associated subgroup analyses to study the effects of age, gender, type of household, and social-economic status on older adults’ perceptions of UGSs. All analyses were performed using SPSS Version 22.0 (IBM SPSS Statistics, New York, NY, USA).

## 3. Results

### 3.1. Respondents’ Characteristics

The overall sample size for the study was 326, with 182 female (56%) and 144 male (44%) respondents. This gender split is approximately matched to the gender demographics in the two cities selected (Table 2). Among the respondents, the largest age group was 75–79 years (19.6%), followed by 80–84 years (16.9%). Approximately 87% of the respondents attained a high-school education or less. Over 70% of the respondents were married and another 17% were widowed. One out of five respondents were older adults living alone, and 37% of the respondents claimed that they had a monthly income lower than US$350 while another 14% had a monthly income somewhere between US$350 and US$500. Our sample skewed higher than general demographics in the selected cities for age (Table 3), with 67% of our sample aged 70 or over. This is expected, given the research question and sampling methodology, where we aimed to collect data from two groups: Younger elderly (55–70) and older elderly (older than 70). For the obtained self-reported health data, our sample’s averaged scores on the PCS and MCS were very close to the results of the 1998 Health Survey in the U.S. provided by the QualityMetric Health Outcomes^TM^ scoring system (±1.5 within 100 scores).

### 3.2. The Planning of Spatial Distribution of UGS

One objective of the questionnaire was to pinpoint the right places for allocating UGS resources to where it is most preferred by older adults [74]. During audits, differences in the popularity of various UGSs were observed. The interviews were then conducted in small-scale UGSs, which are popular among older adults. The average number of older adults present was 20 for all the sites where interviews were conducted, and 90% of the sites had more than 5 elderly visitors present during the interview. The respondents were asked about their frequently visited places near the UGS. Similar answers were obtained from different sites in different districts. On top of the list were wet markets (20% of the respondents), shops (8.2% of the respondents), elderly centers (6% of the respondents), and restaurants (5.5% of the respondents). Such high repeat rates indicate that for older adults, visiting a UGS is closely related to their daily activities in these everyday places. Thus, in terms of site selection for UGSs in the neighborhood, planners should consider geographically incorporating UGSs with older adults’ everyday places.

Due to the decline in mobility in older adults, accessibility is also an important factor to consider in planning the spatial distribution of UGSs. The respondents were asked to estimate the time it takes to walk to the UGS from their residence and whether they consider such a walking distance far or close. The results showed that, for older adults that rated the UGS close or very close, the average walking time was 6.8 min. Given that the comfortable walking speed of older adults is 0.9 to 1.2 m/s [75,76], one can conclude that a green space within 400 m is accessible by walking for older adults.

### 3.3. Usage, Perception of UGSs, and Self-Reported Health

Significant correlations between the UGS usage patterns and self-rated health conditions were observed in the two rounds of surveys conducted in Hong Kong and Tainan. The duration of the UGS visit is positively correlated with the domains of mental health (r = 0.15, *p* = 0.009) and social functioning (r = 0.12, *p* = 0.038) among the 326 responses.

**Figure 4:**
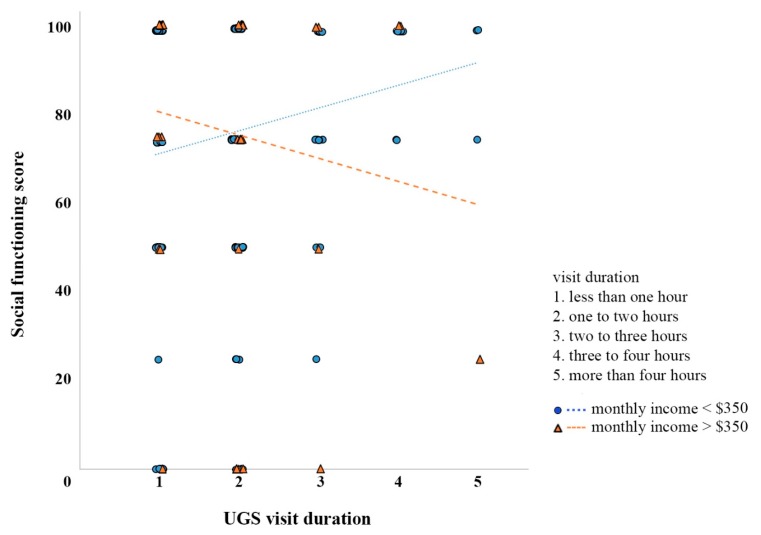
Simple slope analysis of the regression of social functioning scores on UGS visit duration at low and high levels of income.

Income was found to be a significant moderator of the association between the duration of visit and social functioning (b = −3.05, 95% CI [−5.36, −0.74], t = −2.60, *p* = 0.010, (see Figure 4 and Table 4). The relationship was more prominent in respondents with income levels lower than $350 per month (sample mean). Subgroup analysis (see Table 5) also showed that the duration of the visit and social functioning were significantly related among older elderly respondents (*p* = 0.018) but not their younger elderly counterparts (*p* = 0.490). The relationship was also significant among older adults living alone (*p* = 0.035) but not those living with families (*p* = 0.215). Furthermore, the association between the duration of UGS visits and self-rated mental health was significant in female respondents (*p* = 0.016) but not male respondents (*p* = 0.129).

The pilot study showed that perceptions of safety in the UGS were linked to the physical functions of elderly visitors (see Appendix A, Table A1, Table A2 and Table A3). In Hong Kong, perceived safety presents a strong correlation with two subdomains in physical health summary scale in SF-12v2, i.e., the role-physical score (r = 0.28, *p* = 0.002) and the bodily pain score (r = 0.20, *p* = 0.027). On the other hand, for the Tainan data set, perceptions of safety are significantly associated with one role-physical scale item: Accomplished less due to physical health (r = 0.23, *p* = 0.030). After the second-round survey, data from different rounds of surveys were combined to test for trends in the correlations with a larger sample size. Again, perceived safety was positively related to the PCS score (r = 0.12, *p* = 0.037), the domains of physical role (r = 0.17, *p* = 0.002), and bodily pain (r = 0.14, *p* = 0.015). The repeated patterns in different cities and different rounds of surveys suggest that the link between perceived safety and physical health is a robust finding that should be explored further.

**Figure 5:**
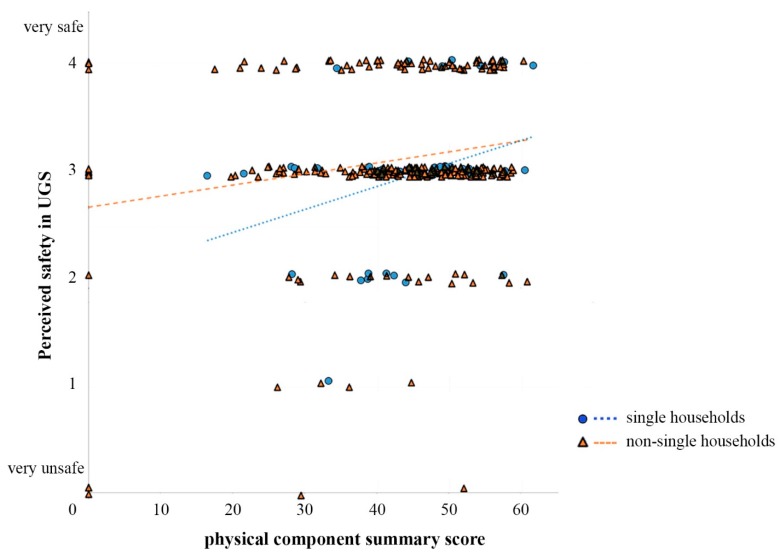
Simple slope analysis of the regression of perceived safety in UGS on PCS (physical component summary) scores by the type of household.

Household type had a significant moderating effect (b = −0.02, 95% CI [–0.038, –0.001], t = −2.02, *p* = 0.044, see Table 6) on the relationship between perceived safety and the PCS score. The relationship was more prominent in respondents living alone (Figure 5). Such results provided further evidence for the relationship between health conditions and perceived safety in the UGS among older adults.

**Figure 6:**
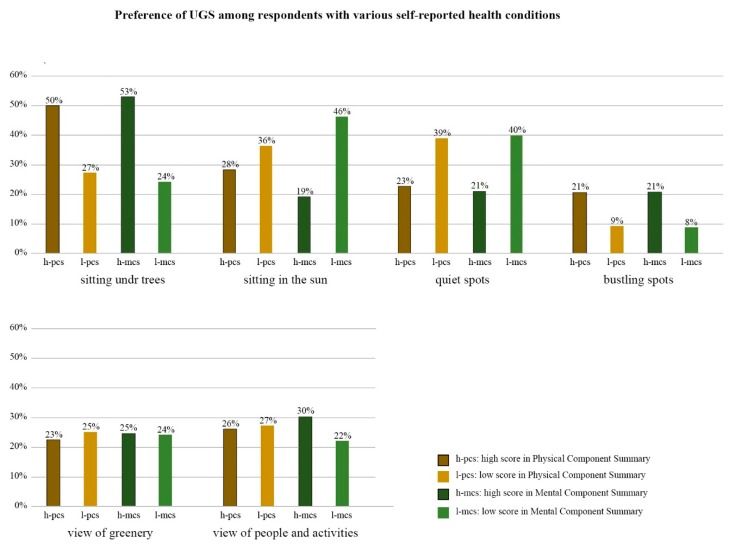
Self-reported health status and preferences for UGS.

Preferences for UGSs were compared between respondents with PCS/MCS scores above and below the sample mean [77,78]. The results of the SF12v2 showed that respondents with PCS/MCS scores above the average preferred to sit under trees and enjoy more lively spots in the UGS (Figure 6). On the other hand, respondents with scores below the average on both components preferred to sit in the sun and enjoy quiet spots in the UGS. Approximately 25% of the respondents favored places with views of greenery regardless of their scores on either component. Respondents with higher MCS scores showed greater preference for seeing other site users and their activities in the UGS than those with lower scores.

### 3.4. Design Aspects and Activities in UGS

This study further demonstrated that perceptions of safety can be influenced by the design of the UGS. There were positive associations between subjective assessment of the aesthetic quality of the UGS and perceived safety (n = 313, r = 0.281, *p* < 0.001, also see the Appendix A Table A2 and Table A3 for consistent evidence from Hong Kong and Tainan). Subgroup analysis found that this association was more significant among older adults with lower incomes (*p* < 0.001) than those with higher oncomes (*p* = 0.120). Multiple linear regression analysis showed that the selected design aspects explained 33% of the variability in the subjective assessment of the aesthetic quality of the UGS. The color of the plants (*p* = 0.048) and maintenance condition (*p* = 0.027) were significant predictors of the subjective assessment of the aesthetic quality. The model revealed that every unit increase in the satisfaction with plant color leads to a 0.23 unit increase in satisfaction with the overall aesthetic quality of the UGS. With the selected design aspects as input variables, the model was significant with r = 0.571, *p* < 0.001 (Table 7). During the interviews, older adults in different sites expressed a preference for a more flower-rich setting than the current UGS.

**Figure 7:**
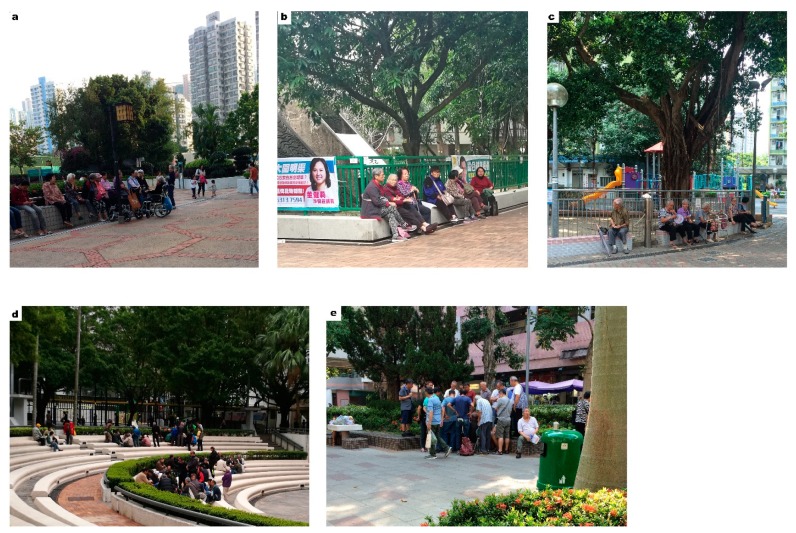
(**a**–**c**) Social interactions of elderly visitors are commonly found in green areas in public housing, but the current design or facilities may not properly facilitate such activities. (**d**,**e**) The most favorite spots for chess players were those that can also house audience and create an atmosphere of gathering. (Photos taken with the knowledge and consent of participants.)

According to the data collected from the second-round interview, older adults’ behavior patterns varied in the different types of UGS. In terms of activities, the highest percentage (36%) of older adult respondents in street parks were passing by and taking a rest. On the other hand, 38% of respondents in green areas in public housing were engaged in a mix of activities (social interactions, physical exercise, etc.) while another 27% were passing by and taking a rest. For visiting companions, approximately 50% of older adults in green areas in public housing claimed that they visit the place with friends. Since public housing generally consists of public rental housing estates that were built by the Hong Kong Housing Authority and subsidized by the Hong Kong Government, tenants generally have a lower socio-economic status and are composed of a large proportion of elderly citizens. Over 70% of respondents in street parks were there by themselves. From both the interviews and site audits, it was noticeable that more social interactions could be found among older adults in the green areas in public housing than in street resting gardens. Design features that can facilitate such social interactions and create an atmosphere of gathering are usually the most popular spots in the UGS (Figure 7).

## 4. Discussion

We examined perceptions of UGSs in elderly people in two cities in Asia, through two rounds of assessment incorporating questionnaires and site surveys. Overall, we found that health aspects, such as mobility, affected elders’ use of and willingness to travel to UGSs. Visiting UGSs had an impact on mental health scores, and subgroups of elders with differing needs were identified based on demographic variables.

Older residents in high-density cities tend to spend considerable amounts of time in UGSs due to overcrowded living environments [10,79]. The results of this study agreed with previous research that a longer UGS visit duration creates positive impacts on older adults’ mental health and social functioning [80,81]. Consistent with previous results [82], this study provides further evidence that potential health benefits associated with UGSs are important to older adults with low social support and social capital, i.e., lower income groups, older elderly groups, and older adults living alone.

Previous studies conducted found that safety concerns are one of the most common reasons for not visiting UGSs in some of American cities [83,84]. No correlations between perceived safety in the UGS and older adults’ usage pattern were found in our study. This could be explained by cultural differences between our sample and previous studies, or the overall perceived crime level in the cities studied. Future studies could be made on the comparison of safety perception in high- and low-risk countries. On the other hand, this study suggests that older adults’ perceptions and preferences for UGSs are influenced by health status. Perceived safety in the UGS showed a positive correlation with several self-reported health indicators (PCS score, physical role, and bodily pain). Such relationships are particularly significant in older adults living alone. These results are in line with several studies indicating that people in poor health or with low social support perceive the environment as more challenging [85,86]. Older adults in poor health and with reduced mobility are less capable of coping with potential safety risks that may occur in public spaces, which contributes to a sense of insecurity [87]. Furthermore, this study supports the findings of Marcus [88] on health status and preferences for physical environments. Older adults with various self-reported health statuses showed different preferences for the settings of UGSs, such as places to stay, views, and atmosphere. Our findings are also consistent with several previous studies of Chinese elderly by Bao [89], Qing [90], and Wei et al. [91] that colorful flowers are appreciated as an aesthetic design element in UGSs. A cultural explanation for such a preference is that bright-colored flowers are linked to the perception of vitality and prosperity among Chinese elderly [92].

Artmann et al. [93] argued that the location of the UGS and the design of greenery elements should consider the mobility constraints of older adults. This study suggests a 400-m walking distance for easy accessibility of UGSs for older adults. Planning of the spatial distribution of UGSs should also take into account older adults’ routine places in the neighborhood. Previous research investigated the relationship between the size and accessibility of UGSs and the levels and amounts of physical activity [94,95]. The differences in the type of activities and companions for elderly visitors in various categories of UGSs, however, has not been well studied [96]. Current practice adopts a universal design for all types of UGSs in the city and does not respond to the behavior patterns of older adults [97]. Based on this study, it was also noticeable that older adults tend to have more social interactions and stay in groups in the UGS in public housing estates than in street resting gardens. Such results suggest different design approaches for different types of UGSs. These results also reiterate the importance of diversity to accommodate the different needs of older adults [98].

The innovation and practical implications of this study are reflected in several ways. First, our interviews were carried out on site, and the respondents were located in the UGS being studied. By establishing such a correspondence, the results were more robust compared to some previous surveys conducted indoors in community centers or clinics [99]. Second, whereas other studies focused on the relationship between self-reported measures of the local environment and health, our study investigated the interactions between usage of UGSs, self-reported health status, and the perceptions and preferences of older adults. Since physical environment and social aspects influence individual types of feelings and behavior; these factors and their interaction needed to be considered in designing age-friendly cities [9]. With the validated SF 12v2 health survey, assessment data on physical health, mental health, and social functioning were included in the analysis. The results comprised the missing evidence necessary to better infer the link between individuals’ perceptions of UGSs and their health or health behaviors [100]. Specific elderly groups (in terms of socio-demographic status) that profit more from UGSs were identified. This study provides timely information for promoting ageing in place, which has become a high priority for governments in an ageing society.

This study focuses on high-density urban settings and small-scale UGSs. High-density is irreversible and becomes normal for city development [101]. Our results indicate that in highly developed urban areas, UGSs tend to be small in size but precious in value for elderly residents in particular [102]. Moreover, studies revealed the increasing popularly of small-scale UGSs among older adults in Asia, Europe, and North America [15,29,38,103]. Our study provides useful information for planning and designing small-scale UGSs and support healthy ageing in a worldwide context.

The limitations of this study include the limited number of neighborhoods in Hong Kong and Tainan, which did not allow pairwise comparisons of the physical characteristics of UGSs. Another potential weakness is the discordance between the subjective and objective assessment of health status that can be found in some cases. [104] pointed out that it might require higher cognitive and physical functioning to complete a short-form health survey than the typical levels found in some groups of older persons. The accuracy of self-reported data may also be limited by other factors, such as the emotional state of the respondents and the environmental settings [105]. For future studies, the design of UGSs for elderly visitors with special needs (recovering, disabled, and health training, etc.) deserves further investigation. A more comprehensive set of case studies from Asian and Western countries is needed to provide better understanding of the aesthetic preferences for UGSs among older adults of different cultures.

## 5. Conclusions

With over 300 samples from two cities, this study aimed to answer three key questions about older adults’ use of UGSs in two cities in Asia. The results showed a potential impact of UGSs on health and wellbeing, where the duration of the visit was positively associated with the social functioning and mental health of older adults, especially those with lower income or single households, older elderly groups, and female respondents. Differences in perceptions of UGSs were found for different health conditions, where perception of safety in the UGS was increased in those with higher physical health scores. Those with higher health scores also preferred more stimulating areas of UGSs (sunny, lively spots with more people), whereas those with lower health scores preferred less stimulating areas (shaded, quiet spots). The key design aspects affecting perception related to the aesthetic qualities of the space, where nicer-looking UGSs were considered more safe. The maintenance condition of the UGS and color of the plants affected perceptions of safety, and older adults preferred to have a greater number of flowers in the UGS. To promote active ageing, UGSs can be situated closer to everyday places, such as wetmarkets, shops, and public housing. In addition, different use cases should be considered for parks based on location, and design features that encourage social interactions should be increased.

## Figures and Tables

**Table 1:** Questionnaire design.

	Investigating Aspect	Detailed Items
1	subjective assessment on quality and characteristics	spaciousness, number of trees, facilities, seating, safety, and aesthetic qualities of UGSs [15]
	usage pattern	time of the day for the visit, visit frequency and duration [52]
	self-reported health status	Short Form-12v2 Health Survey (SF12v2) [49]
	socio-demographic	age, gender, marital status, living arrangement, level of education, income level, and perceived social status [53]
2	accessibility	subjective assessment of the accessibility of UGSs, walking time required from home, obstacles (such as traffic and stairs), and frequently visited places near the UGS [15,54]
	activities in two types of UGSs	visit duration, the type of activities, and companions [55]
	preferences for different designs and settings	e.g., sitting under a tree vs sitting in the sun, view of greenery, visual access to the streetscape, view of other site users and their activities, acoustic environment and atmosphere [56,57]
	design and aesthetic quality	color, shape, and seasonal variation in the vegetation, diversity in species, maintenance, and proportion of soft surfaces [58,59,60]
	perceived safety	reduced visibility associated with dense vegetation, prospect of crime, presence of security guards, fear of falling, and feeling unwell [61].

**Table 2:** Demographic profile of the respondents.

Overall Sample (n = 326)			
**Age**		**Education**	
<59	29 (9%)	Below primary school	68 (21%)
60–69	78 (24%)	Primary school	143 (44%)
70–79	121 (37%)	High school	75 (23%)
>79	98 (30%)	College or above	40 (12%)
**Gender**		**Living Arrangement**	
Female	144 (56%)	Single household	51 (16%)
Male	182 (44%)	Living with families/others	264 (81%)
		Other	11 (3%)
**Marital Status**		**Income Levels**	
Married	246 (75.5%)	<US$250	120 (37%)
Single	10 (3%)	US$250–500	46 (14%)
Widowed	58 (18%)	US$501–1000	36 (11%)
Divorced/separated	10 (3%)	US$1001–1500	29 (9%)
Other	2 (0.5%)	≥US$1500	10 (3%)
		Refused to answer	85 (26%)

**Table 3:** Demographic profile of residents aged over 55 in the two cities (Tainan—End 2017 statistics; Hong Kong—End 2018 statistics).

	Tainan	Hong Kong
**Age** (‘000)		
55–59	151.2 (27%)	644.7 (26%)
60–69	228.4 (41%)	1004 (40%)
70–79	104.6 (19%)	482.9 (19%)
>79	68.2 (12%)	382.6 (15%)
**Gender** (‘000)		
Female	287.7 (52%)	1319.1 (52%)
Male	264.7 (48%)	1195.1 (48%)

**Table 4:** Moderating effect of income on the relationship between UGS (urban green space) visit duration and social functioning score.

**1. Interaction effect of income levels on the relationship between UGS visit duration and social functioning score**						
			*b*	*SE B*	*t*	*p*
**Constant**			58.64	7.76	7.55	*p* < 0.001
Income			5.06	2.27	2.23	*p* = 0.027
UGS visit duration			10.67	3.72	2.87	*p* = 0.005
Income × UGS visit duration			−3.05	1.17	−2.60	*p* = 0.010
*Note: R*^2^ = 0.37						
**2. Conditional effect of UGS visit duration on social functioning score at income level**						
*Income level*	*Effect*	*se*	*t*	*p*	*LLCI*	*ULCI*
<$350	7.62	2.85	2.68	0.008	2.01	13.24
>$350	−3.80	3.35	−1.13	0.258	−10.41	2.81

**Table 5:** Subgroup analysis of the types of households, ages, and genders of the respondents.

**1. Conditional effect of UGS visit duration on social functioning score at household types**						
*Types of households*	*Effect*	*se*	*t*	*p*	*LLCI*	*ULCI*
Single households	9.38	4.42	2.12	0.035	0.68	18.08
Non-single households	2.65	2.13	1.24	0.215	−1.55	6.85
**2. Conditional effect of UGS visit duration on social functioning score at age of respondents**						
Age	*Effect*	*se*	*t*	*p*	*LLCI*	*ULCI*
Under 70 years old	1.76	2.55	0.69	0.490	−3.26	6.78
70 years old or above	6.25	2.63	2.37	0.018	1.07	11.44
**3. Conditional effect of UGS visit duration on mental health score at gender of respondents**						
Gender	*Effect*	*se*	*t*	*p*	*LLCI*	*ULCI*
Male	3.33	2.19	1.52	0.129	−0.98	7.64
Female	4.98	2.06	2.42	0.016	0.93	9.03

**Table 6:** Moderating effect of household type on the relationship between the physical component score (PCS) and perceived safety in the UGS.

**1. Interaction effect of household type on the PCS–perceived safety relationship**						
			*b*	*SE B*	*t*	*p*
**Constant**			0.94	0.83	1.13	0.260
Household type			1.07	0.43	2.48	0.014
PCS			0.04	0.02	2.23	0.027
Household type x PCS			−0.02	0.01	−2.02	0.044
*Note: R*^2^ = 0.36						
**2. Conditional effect of physical component score on perceived safety in UGS at household type**						
*Household Type*	*Effect*	*se*	*t*	*p*	*LLCI*	*ULCI*
Single household	0.021	0.009	2.39	0.018	0.004	0.039
Non-single household	0.002	0.003	0.80	0.423	−0.003	0.008

**Table 7:** Linear model of the subjective assessment of the aesthetic quality of the UGS.

**1. Model summary**					
*R*	*R-square*	*Adjusted R-square*	*SE.*		*Sig.*
0.57	0.33	0.28	0.66		<0.001
**2. Coefficients (Dependent variable: Subjective assessment of aesthetic quality of the UGS)**					
		*b*	*SE B*	*β*	*p*
(Constant)		1.30	0.41	/	0.002
Color of the plants		0.23	0.13	0.24	0.048
Geometry of the plants		0.19	0.12	0.21	0.111
Richness in species		−0.05	0.09	−0.05	0.619
Seasonal variation		0.01	0.07	0.02	0.849
Maintenance		0.23	0.10	0.23	0.027
Proportion of soft surfaces		0.07	0.11	0.07	0.544

## Appendix A

**Table A1:** Response of perception of UGS characteristics (Numbers in brackets are the percentage of respondents).

	Not satisfied at All		Not Satisfied		Satisfied		Very Satisfied	
	HK	TN	HK	TN	HK	TN	HK	TN
Total amount of UGS	0 (0%)	12 (13%)	8 (7%)	10 (11%)	81 (69%)	47 (50 %)	28 (24%)	24 (26%)
Number of trees	8 (7%)	11 (12%)	10 (8%)	20 (22%)	65 (55%)	46 (49%)	35 (30%)	16 (17%)
Amount of exercise facility	2 (2%)	16 (17%)	38 (32%)	27 (29%)	65 (55%)	36 (39%)	13 (11%)	14 (15%)
Amount of seats/resting areas	8 (7%)	11 (12%)	22 (19%)	13 (14%)	63 (53%)	42 (45%)	25 (21%)	27 (29%)
Safety in UGS	2 (2%)	2 (2%)	7 (6%)	14 (15%)	79 (67%)	48 (52%)	30 (25%)	29 (31%)
Aesthetic quality of UGS	0 (0%)	2 (2%)	9 (8%)	16 (17%)	87 (74%)	56 (60%)	21 (18%)	19 (21%)

**Table A2:** Correlation matrix for the characteristics of UGS and health conditions of respondents in Tainan. (** and * indicate significant correlations at 0.01 and 0.05 levels respectively.).

	1	2	3	4	5	6	7	8	9	10	11	12	13
1. Area of UGS	1												
2. Number of trees	0.641 **	1											
3. Amount of facilities	0.714 **	0.650 **	1										
4. Number of seats	0.733 **	0.616 **	0.604 **	1									
5. Perceived safety	0.416 **	0.417 **	0.430 **	0.446 **	1								
6. Aesthetic quality	0.592 **	0.546 **	0.547 **	0.431 **	0.297 **	1							
7. Usage frequency	0.416 **	0.364 **	0.292 **	0.372 **	0.084	0.180	1						
8. Usage duration	0.154	0.156	0.135	0.111	0.054	0.081	0.056	1					
9. Age	0.312 **	0.272 **	0.223 *	0.189	0.082	0.247^*^	0.341 **	−0.085	1				
10. Physical functioning	−0.030	−0.101	0.004	−0.034	0.137	−0.109	−0.158	−0.033	−0.284 **	1			
11. Role physical	0.213 *	0.033	0.110	0.146	0.190	0.108	−0.006	−0.016	−0.074	0.543 **	1		
12. Bodily pain	0.078	−0.087	0.171	0.183	0.148	0.037	−0.041	−0.039	−0.167	0.486 **	0.620 **	1	
13. Role Emotional	0.152	0.109	0.088	0.020	0.104	0.115	−0.048	−0.130	0.165	0.252 *	0.471 **	0.193	1

**Table A3:** Correlation matrix for the characteristics of UGS and health conditions of respondents in Hong Kong. (** and * indicate significant correlations at 0.01 and 0.05 levels respectively.).

	1	2	3	4	5	6	7	8	9	10	11	12	13
1. Area of UGS	1												
2. Number of trees	0.478 **	1											
3. Amount of facilities	0.155	0.121	1										
4. Number of seats	0.393 **	0.238 *	0.434 **	1									
5. Perceived safety	0.078	0.087	0.066	0.086	1								
6. Aesthetic quality	0.148	0.155	0.021	0.010	0.204 *	1							
7. Usage frequency	0.199 *	0.224 *	−0.034	0.000	0.023	0.069	1						
8. Usage duration	0.119	−0.025	−0.162	−0.247 **	−0.141	0.187 *	0.345 **	1					
9. Age	0.029	0.071	−0.093	−0.105	−0.022	0.001	0.256 **	0.069	1				
10. Physical functioning	−0.064	−0.062	−0.062	−0.061	0.168	−0.008	−0.022	−0.103	−0.171	1			
11. Role physical	−0.020	0.064	0.110	−0.024	0.283 **	0.027	0.044	−0.140	−0.076	0.605 **	1		
12. Bodily pain	0.095	0.089	−0.107	0.076	0.204 *	−0.167	0.038	−0.095	−0.143	0.354 **	0.462 **	1	
13. Role Emotional	0.033	−0.086	0.038	−0.023	0.118	−0.025	0.071	−0.046	−0.033	0.308 **	0.551 **	0.297 **	1
